# Evaluation of the Accessibility of Molecules in Hydrogels Using a Scale of Spin Probes

**DOI:** 10.3390/gels8070428

**Published:** 2022-07-08

**Authors:** Iulia Matei, Ana-Maria Ariciu, Elena Irina Popescu, Sorin Mocanu, Alexandru Vincentiu Florian Neculae, Florenta Savonea, Gabriela Ionita

**Affiliations:** 1 “Ilie Murgulescu” Institute of Physical Chemistry of the Romanian Academy, 202 Splaiul Independentei, 060021 Bucharest, Romania; iuliamatei@icf.ro (I.M.); elena.irina.popescu@cn-caragiale.ro (E.I.P.); smocanu@icf.ro (S.M.); aniculae@icf.ro (A.V.F.N.); f.savonea@icf.ro (F.S.); 2 IPG Health Consulting, King Edward Street, Macclesfield SK19 1AQ, UK; ana-maria.ariciu@mccannhealth.com

**Keywords:** polymeric gels, diffusion, EPR spectroscopy, nitroxides

## Abstract

In this work, we explored by means of electron paramagnetic resonance (EPR) spectroscopy the accessibility of a series of spin probes, covering a scale of molecular weights in the range of 200–60,000 Da, in a variety of hydrogels: covalent network, ionotropic, interpenetrating polymer network (IPN) and semi-IPN. The covalent gel network consists of polyethylene or polypropylene chains linked *via* isocyanate groups with cyclodextrin, and the ionotropic gel is generated by alginate in the presence of Ca^2+^ ions, whereas semi-IPN and IPN gel networks are generated in a solution of alginate and chitosan by adding crosslinking agents, Ca^2+^ for alginate and glutaraldehyde for chitosan. It was observed that the size of the diffusing species determines the ability of the gel to uptake them. Low molecular weight compounds can diffuse into the gel, but when the size of the probes increases, the gel cannot uptake them. Spin-labelled Pluronic F127 cannot be encapsulated by any covalent gel, whereas spin-labelled albumin can diffuse in alginate gels and in most of the IPN networks. The EPR spectra also evidenced the specific interactions of spin probes inside hydrogels. The results suggest that EPR spectroscopy can be an alternate method to evaluate the mesh size of gel systems and to provide information on local interactions inside gels.

## 1. Introduction

Hydrogels represent a class of materials that are relevant for diverse applications in the field of cosmetics, in food and medicinal industries, due to the particularity of their solid-like network to sequester a large quantity of water—a biologically relevant liquid. The hydrogel “solid network” can be the result of a chemical or of a physico-chemical assembly of gelators. Most hydrogels result from physically-driven assembly or chemical crosslinking processes of natural or/and synthetic polymers. The main difference between chemical and physical hydrogels is that once the chemical bonds of the chemical network are broken, the gel cannot self-heal, whereas physical hydrogels can be recovered by changing the physical conditions. These materials are able to encapsulate and to release compounds in the water pools delimited by the solid network, and these properties are exploited in a variety of their applications [1,2,3,4,5,6,7,8,9].

The properties of encapsulation and release of compounds entrapped in the hydrogel network are related to the mesh size or correlation length, ξ, which is defined as the average distance between consecutive cross-links [10,11]. The estimation of this parameter can be made by various methods such as solute exclusion, mercury porosimetry, nitrogen adsorption/capillary condensation, microscopy and pulsed field gradient-NMR (PFG-NMR) [12,13,14]. To estimate the mesh size, in some cases, it is necessary to obtain the corresponding xerogel, which means that the determined mesh size can be smaller than that in hydrogel. This parameter provides direct information on the size of the particles that can be entrapped in the hydrogel. 

**Figure 1:**
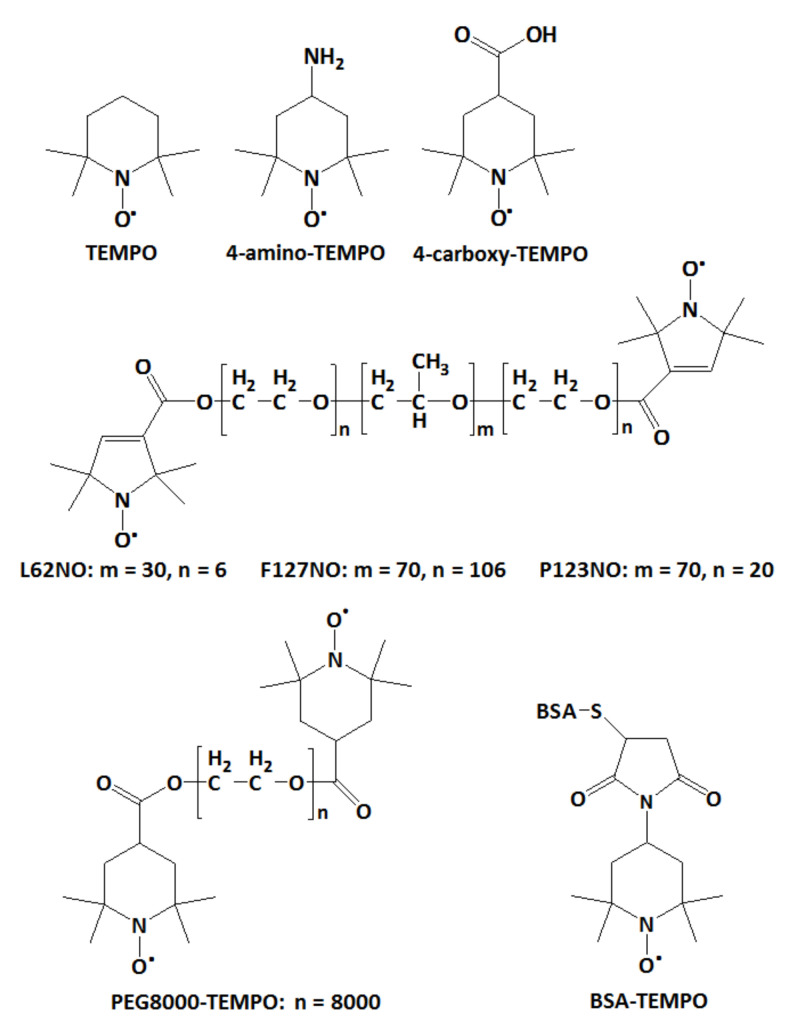
Chemical structures of the spin probes tested for diffusion in hydrogels.

In this study, we provide an alternate method to evaluate the ability of a hydrogel to encapsulate molecules. The electron paramagnetic resonance (EPR) spectroscopy, with its spin probe method, has been used recently by Vesković et al. to determine the water content of hydrogels based on changes in the spectral parameters of PROXYL and TEMPO spin probes [15]. In our study, we use EPR spectroscopy to monitor the diffusion in hydrogels of various spin probes with molecular weights in the range 200–66,000 Da. The spin probes used are depicted in Figure 1 and are of low molecular weight (TEMPO, 4-amino-TEMPO, 4-carboxy-TEMPO, ~200 Da), medium molecular weight (spin-labelled Pluronics L62NO/~2500 Da, P123NO/~5800 Da and F127NO/~12,600 Da; spin-labelled polyethylene glycol, PEG8000-TEMPO/~8000 Da) and high molecular weight (spin-labelled albumin, BSA-TEMPO/~66,000 Da). We analyzed the diffusion of these probes in hydrogels that have polysaccharides and β-cyclodextrins as building blocks of their solid network. 

**Figure 2:**
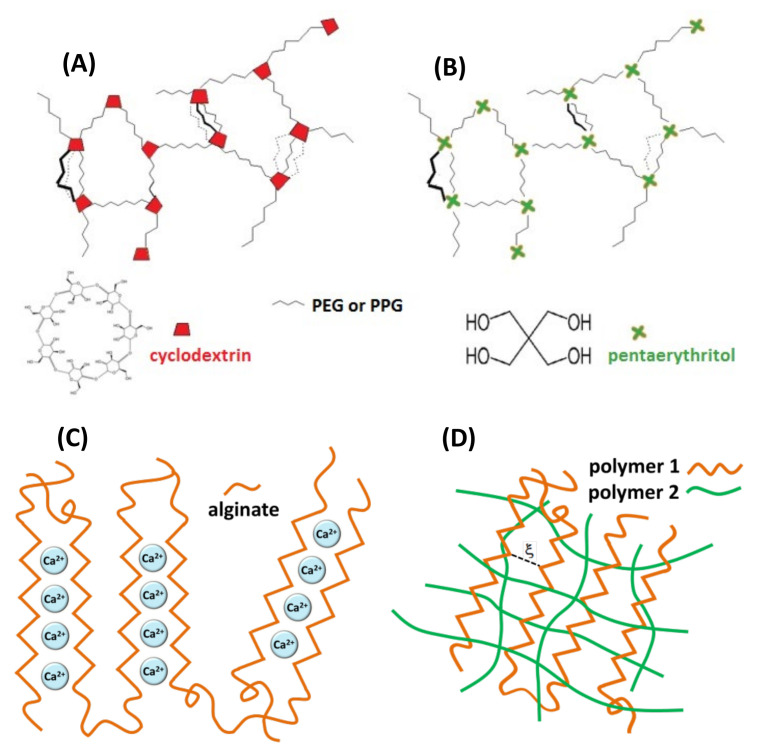
Schematic representation of hydrogel networks: (**A**) covalent gel PEG/β-CD or PPG/β-CD, (**B**) covalent gel pentaerythritol/β-CD, (**C**) ionotropic alginate gel, (**D**) interpenetrating gel network (IPN).

The three types of hydrogels investigated in this study are shown in Figure 2. The first one refers to covalent hydrogel networks formed by reaction of isocyanato-end-capped polyethylene glycol (PEG) or polypropylene glycol (PPG) with β-cyclodextrin (β-CD) using poly glycol chains of different length and different poly glycol:β-CD initial ratios, as described previously [16]. The following covalent hydrogels containing cyclodextrins were prepared: PEG900/β-CD (4:1), PEG900/β-CD (10:1), PEG900/β-CD (14:1), PEG2000/β-CD (10:1) and PPG2000/β-CD (10:1). A covalent gel in which β-CD was replaced by pentaerythritol in the nodes of the network was also prepared, in order to demonstrate that host–guest complexation with β-CD cavities in the gel influences the dynamics of the spin probe. Alginate gel and IPN or semi-IPN gel networks were formed by adding crosslinking agents to alginate/chitosan solutions (Ca^2+^ for alginate and glutaraldehyde for chitosan). 

## 2. Results and Discussion

For all hydrogels, in order to evidence the diffusion of each spin probe, a certain quantity of hydrogel (e.g., 500 mg) was immersed in 1 mL aqueous solution of spin probe (5 × 10^−4^ M), and the system was kept overnight for equilibration. After this, the EPR signal was checked both in solution (termed supernatant) and in gel. In the aqueous solution, nitroxide spin probes have isotropic or quasi-isotropic motion (Supplementary Materials, Figure S1). Compared with solution, in the gel systems considered in this study, the encapsulation of nitroxides is often accompanied by specific interactions of the probes inside the gel, and their distribution in the non-uniform compartments of the gel. From this point of view, EPR spectroscopy is a suitable method to investigate such systems as the EPR parameters of the spin probes (hyperfine coupling constant, a_N_, and rotational correlation time, τ) that are sensitive to changes of dynamics and to the polarity of the microenvironment [17]. The interactions of spin probes inside the gel can be translated into changes in dynamics from an isotropic motion to a highly restricted one, which are reflected in the shape of the EPR spectra [18].

Covalent hydrogels are good examples to highlight the non-homogeneity in gel systems, as they consist of solvent pools in which the spin probe can move freely, and cyclodextrin cavities that can accommodate spin probes fully or partially. In addition, spin probes can interfere with the fiber network of the gel. The diffusion of TEMPO, 4-amino-TEMPO and 4-carboxy-TEMPO in all covalent hydrogels considered in this study shows an unchanged spin probe dynamic as compared to solution. It is known that TEMPO-type radicals form complexes with cyclodextrins in solution, as proven by the slight variation in dynamics and the decrease in the a_N_ value, although the binding constants are relatively low, in the range 10^2^–10^3^ M^−1^ [19,20,21]. Differently, encapsulation of adamantyl-TEMPO [16] or of dual molecular probes bearing a pyrene or dansyl fluorophore and a TEMPO paramagnetic moiety [22,23] determines changes in the EPR spectra of these probes, proving the non-homogeneity of these covalent hydrogels; the presence of two spectral components was observed, corresponding to an equilibrium between the free probe located in the water pools of the gel, and the probe complexed with β-CDs from the network nodes. In the case of the low molecular weight TEMPO radicals used in this study, the fact that their dynamic is not changed after encapsulation, revealing only one component with EPR parameters similar to the solution, indicates that these probes are not complexed by β-CD cavities, but are located in the water pools delimited by the solid network of the gels. This might be a surprising result compared to those reported in the case of adamantyl-TEMPO [16] or dual molecular probes [22,23]. However, we have to take into account that the binding constants for adamantyl derivatives and dansyl probes are 2–3 orders of magnitude higher than those of TEMPO derivatives, and this can explain the differences.

In the case of polymeric spin probes (L62NO, P123NO, F127NO and PEG8000-TEMPO), a different behavior is observed as the molecular weight of the probe increases. Thus, L62NO and P123NO diffuse in all covalent gels, whereas F127NO and PEG8000-TEMPO are only encapsulated by some of the gels. Table S1 summarizes the ability of covalent gels to selectively uptake polymeric spin probes. It can be observed that, in the case of polymeric probes with molecular weight around 10,000 Da, the accessibility in these gels is difficult or does not occur. In the previous EPR study regarding the influence of the ratio between the number of PEG chains and β-CD units on the gel properties, it was observed that, as this ratio increases, the gel network becomes less rigid [16]. Therefore, for PEG900/β-CD (14:1), the diffusion of probes with molecular weight around 10,000 Da was not observed. This has been proven by the fact that only the supernatant has an EPR signal (Figure S2).

Increasing the gel fiber length by increasing the number of PEG units from 900 to 2000 made possible the diffusion of the polymeric probes F127NO and PEG8000-TEMPO in the PEG2000/β-CD gel (Table S1). Replacing PEG2000 with PPG2000, which in fact reduces the fiber length, led once again to the impossibility of diffusion for F127NO and PEG8000-TEMPO. It was also observed that the PPG2000/β-CD hydrogel can uptake completely spin probes with low molecular weight, whereas in the case of polymeric spin probes, a distribution between gel and supernatant ensued.

**Figure 3:**
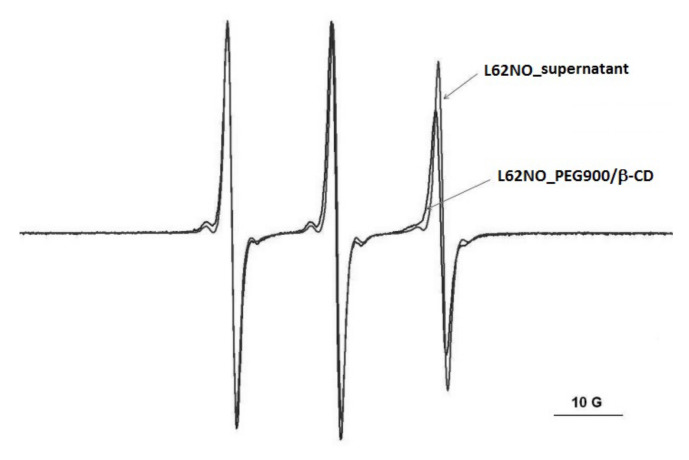
The EPR spectra of L62NO in PEG900/β-CD (10:1) hydrogel and in supernatant, after equilibration.

In the case of the L62NO spin probe, partial diffusion takes place in the PEG900/β-CD (10:1) hydrogel (Figure 3). The same behavior was observed for the P123NO spin probe (Figure S3). For P123NO, the spectra in water, supernatant and gel are presented. The EPR signals are similar, which means that the formation of micelles of these block copolymers does not take place inside the gel.

**Figure 4:**
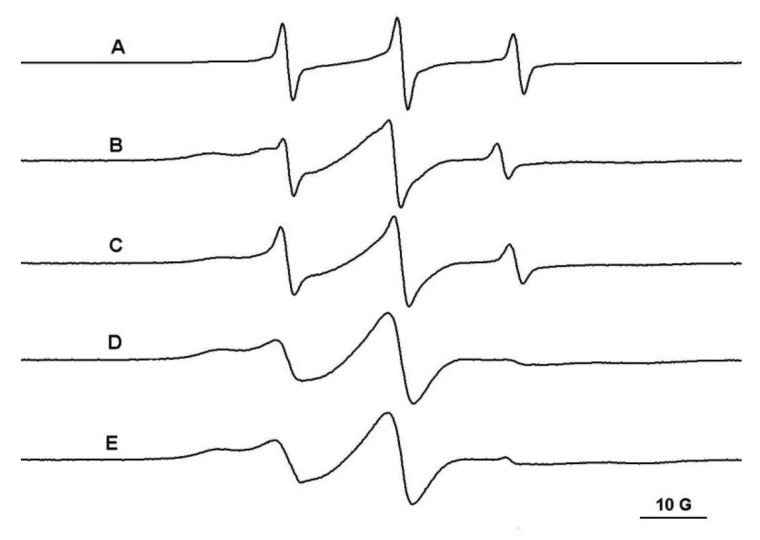
The EPR spectra of TEMPO (**A**), L62NO (**B**), P123NO (**C**), PEG8000-TEMPO (**D**) and F127NO (**E**) encapsulated in PEG900/β-CD (4:1) hydrogel.

The PEG/β-CD covalent hydrogels have different mesh sizes, which depends on the initial ratio of reactants. Thus, in the case of the PEG900/β-CD (4:1) hydrogel, it was observed that the probes with molecular weight in the range 200–12,600 Da diffuse into the gel (Figure 4). Moreover, in the case of polymeric probes encapsulated in covalent hydrogels, the EPR spectra exhibit a two-component feature, with one component presenting a restricted motion and another component corresponding to a faster dynamic.

**Figure 5:**
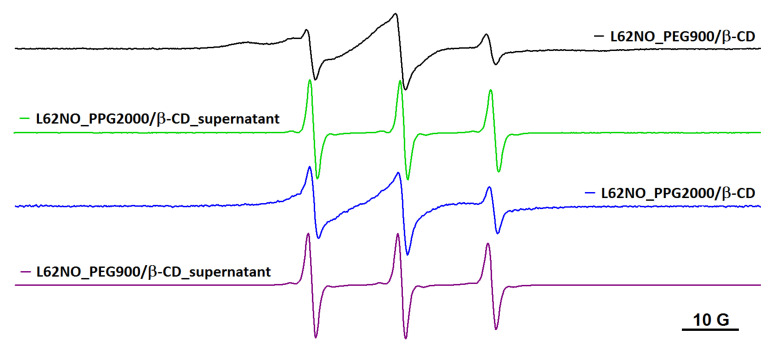
The EPR spectra of L62NO in PEG900/β-CD (4:1), PPG2000/β-CD (10:1) and corresponding supernatant solutions, after equilibration.

The fact that the spin probe motion inside the hydrogel depends on the length of the chain bridging adjacent cyclodextrin units is suggested in Figure 5 for the case of L62NO. In this figure are shown the EPR spectra in PEG900/β-CD (4:1) and PPG2000/β-CD (10:1), and in the corresponding supernatants after equilibration. It can be noted that the immobilized component contribution is less evident in the PPG2000/β-CD hydrogel. The EPR spectra of L62NO encapsulated in PEG900/β-CD (10:1) and PEG2000/β-CD (10:1) are similar to the spectrum of L62NO in water. These results are in accordance with those previously reported, which show that the rigidity and, to some extent, the ability of these systems to encapsulate molecules depend on the PEG/β-CD ratio [16].

**Figure 6:**
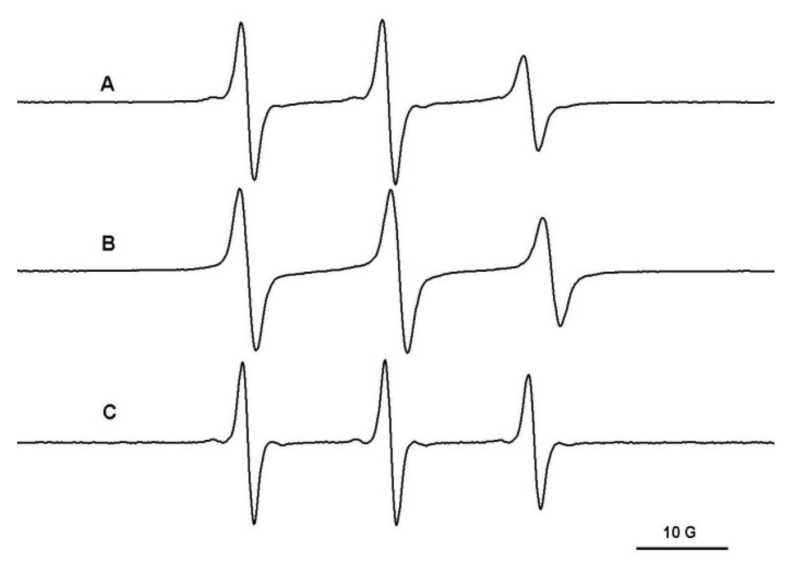
The EPR spectra of L62NO (**A**), P123NO (**B**) and F127NO (**C**) in PEG900/pentaerythritol hydrogel.

The immobilized component of polymeric spin probes in PEG/β-CD hydrogels can be assigned to the complexation of these probes by the cyclodextrins placed in the nodes of the gel network. To prove this, we monitored the diffusion in a hydrogel in which cyclodextrins were replaced with pentaerythritol units. In this case, the immobilized component is not observed (Figure 6). 

In what concerns spin-labelled BSA, this probe was not encapsulated by any covalent hydrogel. This has already been shown in the case of PEG900/β-CD (10:1), in a study reporting the ability of this hydrogel to strip sodium dodecyl sulphate (SDS) molecules from their complex with human serum albumin (HSA) [24]. In that study, it was proved that although SDS micelles have a similar diameter to HSA [25,26], SDS micelles are removed from the surface of HSA by diffusion of SDS into the gel at the molecular level, followed by reorganization into micelles inside the water pools of the hydrogel. Moreover, another study regarding the formation of gold nanoparticles inside the PEG900/β-CD hydrogel showed that nanoparticles with the size of 2–5 nm cannot be released from this gel [27]. This recommended the PEG900/β-CD hydrogel/gold nanoparticles hybrid material for its catalytic ability to neutralize 4-nitrophenol [28].

**Figure 7:**
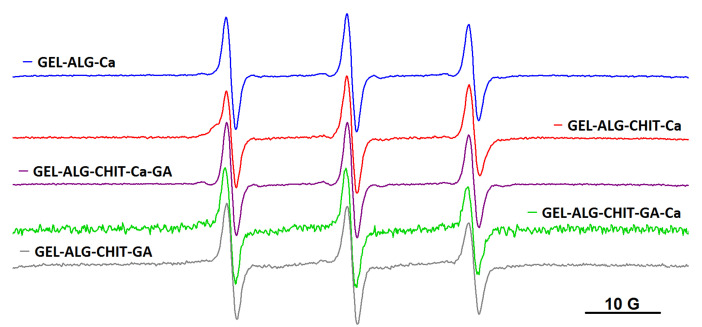
The EPR spectra of F127NO in alginate, semi-IPN and IPN alginate/chitosan hydrogels.

As it was mentioned in Introduction, other polysaccharide-based hydrogels were explored as well: the ionotropic gel resulted by complexation of alginate with Ca^2+^ ions (denoted ALG_Ca), the semi-IPN networks formed either by adding Ca^2+^ to a solution of alginate and chitosan (ALG_CHIT_Ca) or by adding glutaraldehyde (GA) to a solution of alginate and chitosan (ALG_CHIT_GA) and the IPN gels formed by adding Ca^2+^ first and GA second to a solution of alginate and chitosan (ALG_CHIT_Ca_GA) or by adding glutaraldehyde first and Ca^2+^ second to a solution of alginate and chitosan (ALG_CHIT_GA_Ca). Diffusion experiments in these hydrogels revealed that all spin probes are encapsulated by polysaccharide gels, with no changes occurring in the dynamics irrespective of the molecular weight of the spin probe. Thus, all spin probes obtained by spin labelling block copolymers F127, L62 and P123 diffused into alginate gel and into semi-IPN and IPN alginate/chitosan hydrogels, as exemplified in Figure 7 for the case of F127NO. The EPR parameters of the spin probes in these gels are given in Table S2.

**Figure 8:**
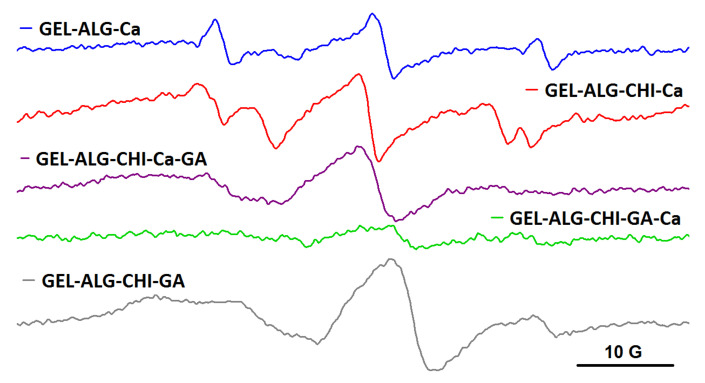
The EPR spectra of BSA-TEMPO in alginate, semi-IPN and IPN hydrogels.

A different behavior was noted for the spin-labelled albumin probe, BSA-TEMPO. The EPR measurements demonstrated that the semi-IPN and IPN systems resulted by adding crosslinking agents have a different capacity to entrap BSA-TEMPO, depending on the order in which the crosslinking agents are added. As it can be observed from Figure 8, BSA-TEMPO diffuses in the alginate gel (ALG_Ca), showing a free motion that indicates no interaction between BSA-TEMPO and the gel fibers. This can be explained by taking into consideration the fact that the BSA surface is negatively charged in our working conditions [29], and that the alginate gel also presents free carboxylate groups. These groups belong mainly to the D-mannuronate residues, as it is known that Ca^2+^ ions bind preferentially to the L-guluronate residues of alginate [30]. The literature data show that the alginate gel can accommodate large macromolecules such as proteins [31] and DNA [32], and even cells [33]. However, in the case of semi-IPN networks, the diffusion experiments show different behaviors. When only Ca^2+^ is added (ALG_CHIT_Ca), the structure of the semi-IPN network is largely that of the alginate gel. The EPR spectrum reveals a two-component feature, with one component similar to the one in alginate gel and another revealing a more restricted motion. The latter component arises from an interaction between BSA-TEMPO and the amino groups present in the structure of chitosan. In the case of the semi-IPN network obtained by adding only GA to the alginate/chitosan solution (ALG_CHIT_GA), BSA-TEMPO diffuses in the gel and shows a highly restricted motion, which can be explained by a strong interaction of BSA-TEMPO with the free amino groups in the chitosan gel fibers. 

The mesh size of IPN hydrogels, and thus the ability of the hydrogels to encapsulate large molecules such as spin-labelled BSA, depends on the order in which the two crosslinkers are added. As it can be observed in Figure 8, BSA-TEMPO diffuses and has a restricted motion in the IPN formed by adding Ca^2+^ first and then GA (ALG_CHIT_Ca_GA), whereas in the case of the IPN formed by adding first GA and then Ca^2+^ (ALG_CHIT_GA_Ca), diffusion hardly occurs and the EPR signal becomes very weak. The spectral features can be rationalized as follows. In the ALG_CHIT_Ca_GA system, the less dense alginate gel network is formed first, followed by grafting of the chitosan network. The interaction between BSA-TEMPO and the remaining free amino groups in chitosan can be observed. In the case of the ALG_CHIT_GA_Ca system, the addition of Ca^2+^ to the ALG_CHIT_GA semi-IPN determines an increase in the density of the gel fibers that has as effect the decrease in the mesh size of the IPN network. As a consequence, the capacity of BSA-TEMPO to diffuse in this IPN is significantly reduced. Thus, we can conclude that, for the IPN hydrogel resulting from the successive addition of GA and Ca^2+^ to the alginate/chitosan solution, the mesh size is smaller than the size of BSA-TEMPO.

## 3. Conclusions

To summarize, we have shown that by using EPR spectroscopy, it was possible to obtain information on the encapsulation properties of a series of polymeric gels obtained by different methods, and to correlate in some cases with the mesh size of these systems. We observed that, in the case of the covalent gels containing cyclodextrin, the encapsulation depends on the length of the polymer linkers and also involves complexation by cyclodextrins. In the case of small mesh sizes, the spin probes cannot penetrate the gel network and no EPR signal appears in the gel samples. In the case of large meshes with strong interactions, an EPR spectrum appears, indicating a restricted or strongly restricted motion. Experiments have shown that polymer gels have a mesh size of less than 10 nm (BSA size) compared to alginate and to IPN hydrogels with chitosan. The IPN and semi-IPN networks of alginate and chitosan have the mesh size greater than the size of BSA, and also exhibit interaction with this protein. The EPR spectroscopy is a suitable method for obtaining information on interactions inside hydrogel networks involving encapsulated molecules, and can also provide information on the mesh size.

## 4. Materials and Methods

Polyethylene glycols PEG900, PEG2000, PPG2000 and β-CD were supplied by Sigma-Aldrich (St. Louis, MO, USA) and Alfa Aesar (Ward Hill, MA, USA). Hexamethylene diisocyanate (HDMI), dibutyltin dilaurate, pentaerythritol (2,2-bis(hydroxymethyl)-1,3-propanediol), dimethylformamide (DMF), TEMPO, 4-amino-TEMPO and 4-carboxy-TEMPO were purchased from Aldrich. Sodium alginate (very low viscosity) and chitosan (low molecular weight) were from Alfa Aesar and Aldrich, respectively.

The covalent gels formed by reaction of PEG900, PEG2000 or PPG2000 with β-CD are referred to as PEG900/β-CD, PEG2000/β-CD and PPG2000/β-CD. The gels were prepared according to the procedure reported by Cesteros et al. [34,35]. Before reacting, the glycols and cyclodextrin were dehydrated. PEGs and PPG were functionalized by reaction with HDMI in DMF, and after that the β-CD solution was added dropwise to the reaction mixture, as reported earlier [27]. The gelation was completed in a few days. For PEG900/β-CD hydrogels, the PEG900 to β-CD ratio was 10:1, 14:1 or 4:1. In the case of PEG2000/β-CD and PPG2000/β-CD, the ratio was 10:1.

The polymeric spin probes L62NO, F127NO, P123NO and PEG8000-TEMPO were obtained following the procedures indicated in the literature [36]. Spin-labelled BSA, BSA-TEMPO, was obtained as described by Feix et al. [37].

For all diffusion experiments, 25 μL of spin probe stock solution (2 × 10^−2^ M in ethanol) was evaporated, then the spin probe was redissolved in 1 ml water in which 0.5 g of hydrogel was left overnight to equilibrate. 

The EPR spectra were recorded on an X-band spectrometer FA100 from JEOL (Tokyo, Japan) at room temperature using the following settings: frequency modulation of 100 kHz, microwave power of 0.998 mW, sweep time of 480 s, modulation amplitude of 1 G, time constant of 0.3 s and a magnetic field scan range of 100 G.

## Supplementary Materials

The following supporting information can be downloaded at: https://www.mdpi.com/article/10.3390/gels8070428/s1, Figure S1: The EPR spectra of the nitroxide-type spin probes used for diffusion experiments in hydrogels; Table S1: Encapsulation of polymeric spin probes in covalent hydrogels; Figure S2: The EPR spectra of F127NO in PEG900/β-CD (1:10) hydrogel and in supernatant, after equilibration; Figure S3: The EPR spectra of P123NO in water, in PEG900/β-CD (10:1) hydrogel and in supernatant, after equilibration; Table S2: Hyperfine coupling constants (a_N_) and rotational correlation times (τ) of spin probes in alginate, semi-IPN and IPN hydrogels.
