# Supplementary material for: Evaluation of the Accessibility of Molecules in Hydrogels Using a Scale of Spin Probes
Source: Gels. 2022 Jul 8;8(7):428. doi: 10.3390/gels8070428 (PMC9316983; doi:10.3390/gels8070428)
Supplement: Supplementary file 1 [file gels-08-00428-s001.zip › gels-1794125-supplementary.pdf]

Supplementary Material

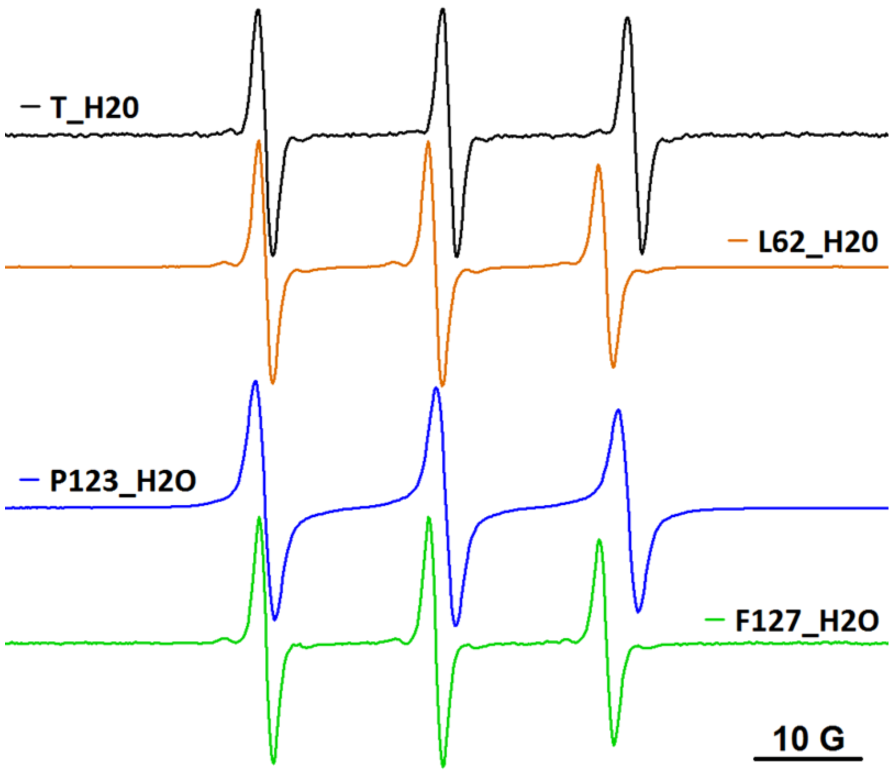

Figure S1. The EPR spectra of the nitroxide-type spin probes used for diffusion experiments in hydrogels.

Table S1. Encapsulation of polymeric spin probes in covalent hydrogels

Gel	TEMPO	L62NO	P123NO	F127NO	PEG8000-TEMPO
PEG900/β-CD (10:1)	Yes	Yes	Yes	No	No
PEG900/β-CD (4:1)	Yes	Yes	Yes	Yes	Yes, signal low
PEG900/β-CD (14:1)	Yes	Yes	Yes	No	No
PEG2000/β-CD (10:1)	Yes	Yes	Yes	Yes, signal low	Yes, signal low
PPG2000/β-CD (10:1)	Yes	Yes	Yes	No	No

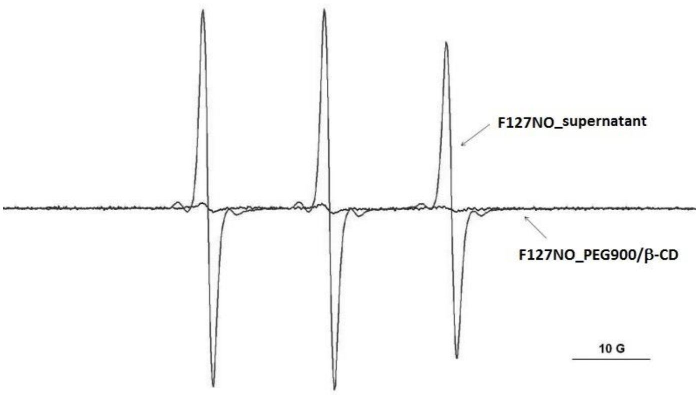

Figure S2. The EPR spectra of F127NO in PEG900/β-CD (10:1) hydrogel and in supernatant, after equilibration.

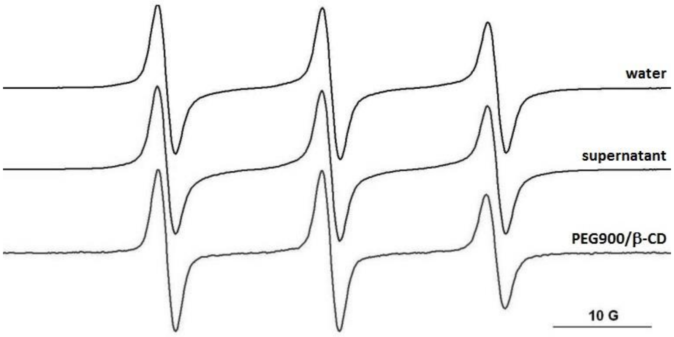

Figure S3. The EPR spectra of P123NO in water, in PEG900/β-CD (10:1) hydrogel and in supernatant, after equilibration.

Table S2. Hyperfine coupling constants (a_N) and rotational correlation times (τ) of spin probes in alginate, semi-IPN and IPN hydrogels

**ALG_Ca**	**$2a_N$ (G)**	**a_N (G)**	**ΔH_0 (G)**	**h_{-1}**	**h_0**	**h_{+1}**	**$\tau \times 10^{10}$ (s)**
4-amino-TEMPO	33.86	16.93	1.75	1.80	1.80	1.59	0.75
4-carboxy-TEMPO	34.20	17.10	1.71	1.90	1.90	1.60	0.99
L62NO	31.99	16.00	1.34	1.54	1.95	1.37	2.74
F127NO	31.99	16.00	1.33	1.81	1.91	1.57	1.14
P123NO	32.01	16.01	1.33	1.64	1.90	1.50	1.77
**ALG_CHIT_Ca**	**$2a_N$ (G)**	**a_N (G)**	**ΔH_0 (G)**	**h_{-1}**	**h_0**	**h_{+1}**	**$\tau \times 10^{10}$ (s)**
4-amino-TEMPO	33.85	16.93	1.77	1.90	1.92	1.73	0.68
4-carboxy-TEMPO	34.38	17.19	1.76	1.87	1.88	1.68	0.70
L62NO	31.91	15.96	1.41	1.78	1.90	1.52	1.40
F127NO	32.02	16.01	1.37	1.57	1.90	1.48	2.10
P123NO	32.07	16.04	1.40	1.41	1.98	1.40	3.43
**ALG_CHIT_Ca_GA**	**$2a_N$ (G)**	**a_N (G)**	**ΔH_0 (G)**	**h_{-1}**	**h_0**	**h_{+1}**	**$\tau \times 10^{10}$ (s)**
4-amino-TEMPO	33.87	16.94	1.79	1.87	1.92	1.69	0.91
4-carboxy-TEMPO	34.39	17.20	1.64	1.91	1.92	1.72	0.66
L62NO	31.89	15.95	1.31	1.74	1.85	1.49	1.24
F127NO	31.90	15.95	1.25	1.51	1.55	1.25	1.05
P123NO	31.84	15.92	1.32	1.62	1.83	1.44	1.63
**ALG_CHIT_GA_Ca**	**$2a_N$ (G)**	**a_N (G)**	**ΔH_0 (G)**	**h_{-1}**	**h_0**	**h_{+1}**	**$\tau \times 10^{10}$ (s)**
4-amino-TEMPO	33.60	16.80	1.96	1.37	1.99	1.07	0.73
4-carboxy-TEMPO	34.28	17.14	1.78	1.80	1.95	1.57	1.78
L62NO	31.83	15.92	1.18	1.75	1.80	1.36	1.26
F127NO	31.93	15.97	1.39	1.90	1.93	1.44	1.53
P123NO	31.79	15.90	1.35	1.60	1.80	1.44	1.61
**ALG_CHIT_GA**	**$2a_N$ (G)**	**a_N (G)**	**ΔH_0 (G)**	**h_{-1}**	**h_0**	**h_{+1}**	**$\tau \times 10^{10}$ (s)**
4-amino-TEMPO	33.80	16.90	1.76	1.90	1.97	1.72	0.97
4-carboxy-TEMPO	34.34	17.17	1.82	1.96	1.99	1.53	1.76
L62	31.88	15.94	1.33	1.86	1.94	1.38	1.79
F127	31.83	15.92	1.37	1.89	1.91	1.48	1.24
P123	31.71	15.86	1.41	1.66	1.78	1.25	2.09

Note: Rotational correlation times, τ, were calculated using the equation:

$$\tau_c = 6.51 \times 10^{-10} \Delta H_0 \left[\left(\frac{h_0}{h_{-1}} \right)^{1/2} + \left(\frac{h_0}{h_{+1}} \right)^{1/2} - 2 \right]$$

where ΔH_0 is the peak-to-peak width (in Gauss) of the central line, and h_{-1}, h_0 and h_{+1} are the heights of the low, central and high field lines, respectively [Stones, T.J.; Buckman, T.; Nordio, P.L.; McConnell, H.M. Spin-labeled biomolecules. *Proc. Natl. Acad. Sci. U.S.A.* **1965**, *54*, 1010–1017. doi: 10.1073/pnas.54.4.1010].
